# Supplementary material for: Modulation of viral replication, autophagy and apoptosis by induction and mutual regulation of transcription factors EB and E3 during coronavirus infection
Source: Front Microbiol. 2025 Dec 10;16:1609598. doi: 10.3389/fmicb.2025.1609598 (PMC12727985; doi:10.3389/fmicb.2025.1609598)
Supplement: Supplementary file 1 [file Data_Sheet_1.docx]

**Supplementary figures Figure Legends**

**Fig. S1**

**Effects of TFEB overexpression on the replication of IBV.**

DF1 cells were transfected with pXJ40-Flag and pXJ40-Flag-TFEB, respectively. At 24 hours post-transfection, cells were either infected with IBV at an MOI~2 or treated with UV-inactivated IBV, harvested at the indicated time points and subjected to Western blot analysis using the indicated antibodies. Beta-actin was included as the loading control.

# Fig. S2

**Regulation of viral replication, autophagy and apoptosis by TFEB and TFE3 during HCoV-OC43 infection.**

(A) The stable TFE3-knockdown H1299 cell clones were infected with HCoV-OC43 at an MOI∼2, harvested at the indicated time points and subjected to Western blot using the indicated antibodies. Percentage of PARP cleavage [PARP Clv. (%)] was shown as the intensity of cleaved PARP (Cl) divided by the total intensities of the full-length PARP (FL) + Cl.(B) The stable TFE3-knockdown H1299 cell clones were infected and harvested as described in (A). Total RNA was extracted and mRNA levels of TFEB, HCov-OC43 gRNA, SQSTM1 and CHOP were determined by RT-qPCR (ns, non-significant; *, P < 0.05; **, p < 0.01; ***, p < 0.001).

# Fig. S3

**Regulation of viral replication, autophagy and apoptosis by TFEB and TFE3 during PEDV infection.**

A .The stable TFE3-knockdown H1299 cell clones were infected with PEDV at an MOI∼2, harvested at the indicated time points and subjected to Western blot using the indicated antibodies. Percentage of PARP cleavage [PARP Clv. (%)] was shown as the intensity of cleaved PARP (Cl) divided by the total intensities of the full-length PARP (FL) + Cl. B. The stable TFE3-knockdown H1299 cell clones were infected and harvested as described in (A). Total RNA was extracted and mRNA levels of TFEB, PEDV gRNA, SQSTM1 and CHOP were determined by RT-qPCR (ns, non-significant;

*, P < 0.05; **, p < 0.01; ***, p < 0.001).

# Fig. S4

**The effect of TFE3 overexpression in TFE3-knockdown H1299 cells infected with IBV on the regulation of IBV replication, autophagy, and apoptosis.**

A. The stable H1299-shTFE3 cells were transfected with pXJ40-Flag and pXJ40-Flag-TFE3, respectively. and were either infected with IBV at an MOI~2 or treated with UV-inactivated IBV at 24 h post-transfection. Cells were harvested at the indicated time points and subjected to Western blot analysis using the indicated antibodies. Beta-actin was included as the loading control. B. Total RNA was extracted from IBV-infected stable H1299-shTFE3 cells described in (A), the level of IBV-gRNA and the mRNA levels of TFE3, SQSTM1, CHOP, MCLON1 and CTSB were determined by RT-qPCR (ns, non-significant; *, P < 0.05; **, p < 0.01; ***, p < 0.001).
