# Supplementary figures and images for: Modulation of viral replication, autophagy and apoptosis by induction and mutual regulation of transcription factors EB and E3 during coronavirus infection
Source: Front Microbiol. 2025 Dec 10;16:1609598. doi: 10.3389/fmicb.2025.1609598 (PMC12727985; doi:10.3389/fmicb.2025.1609598)

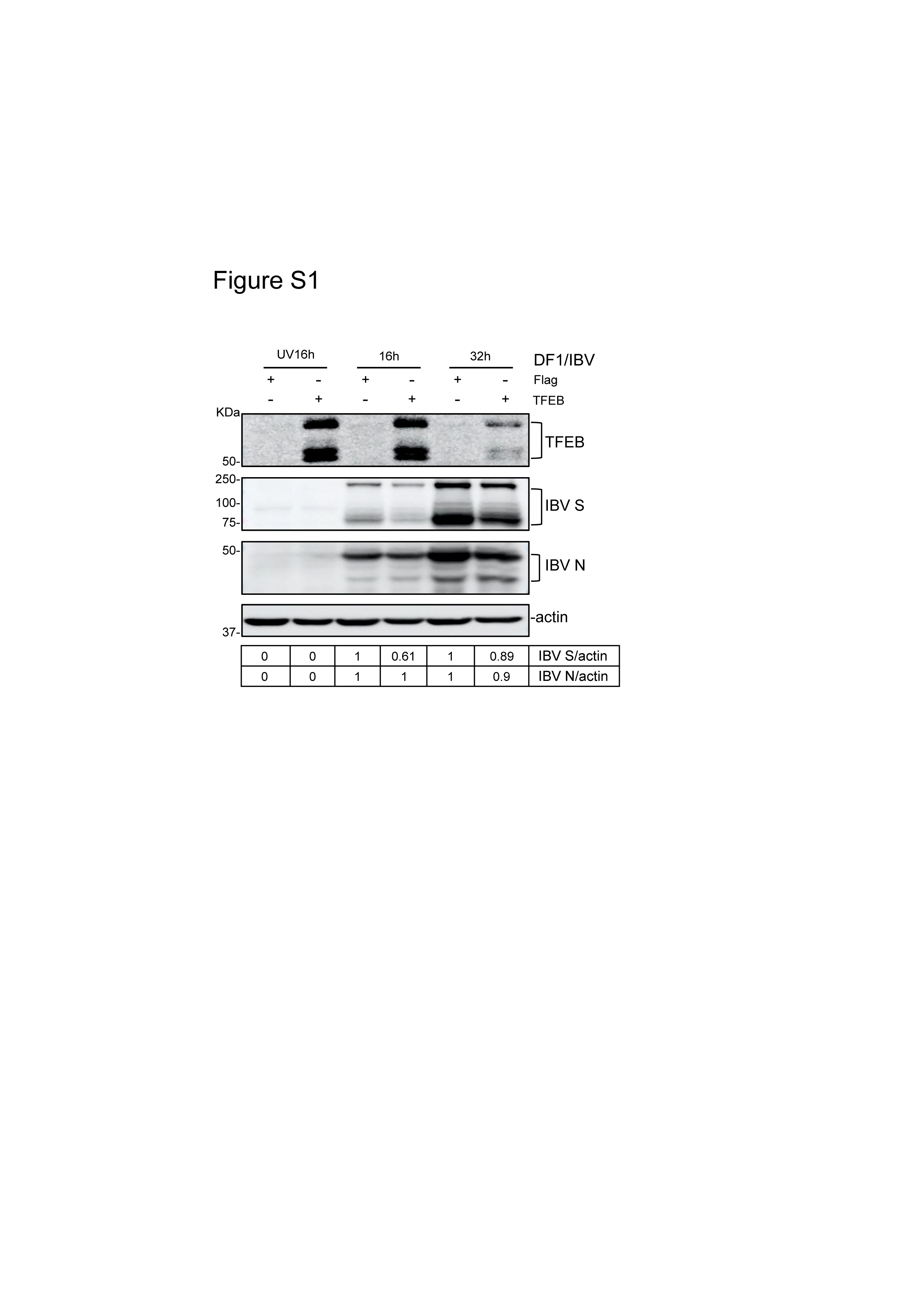

Supplement: Supplementary file 2 [file Image_1.tif]

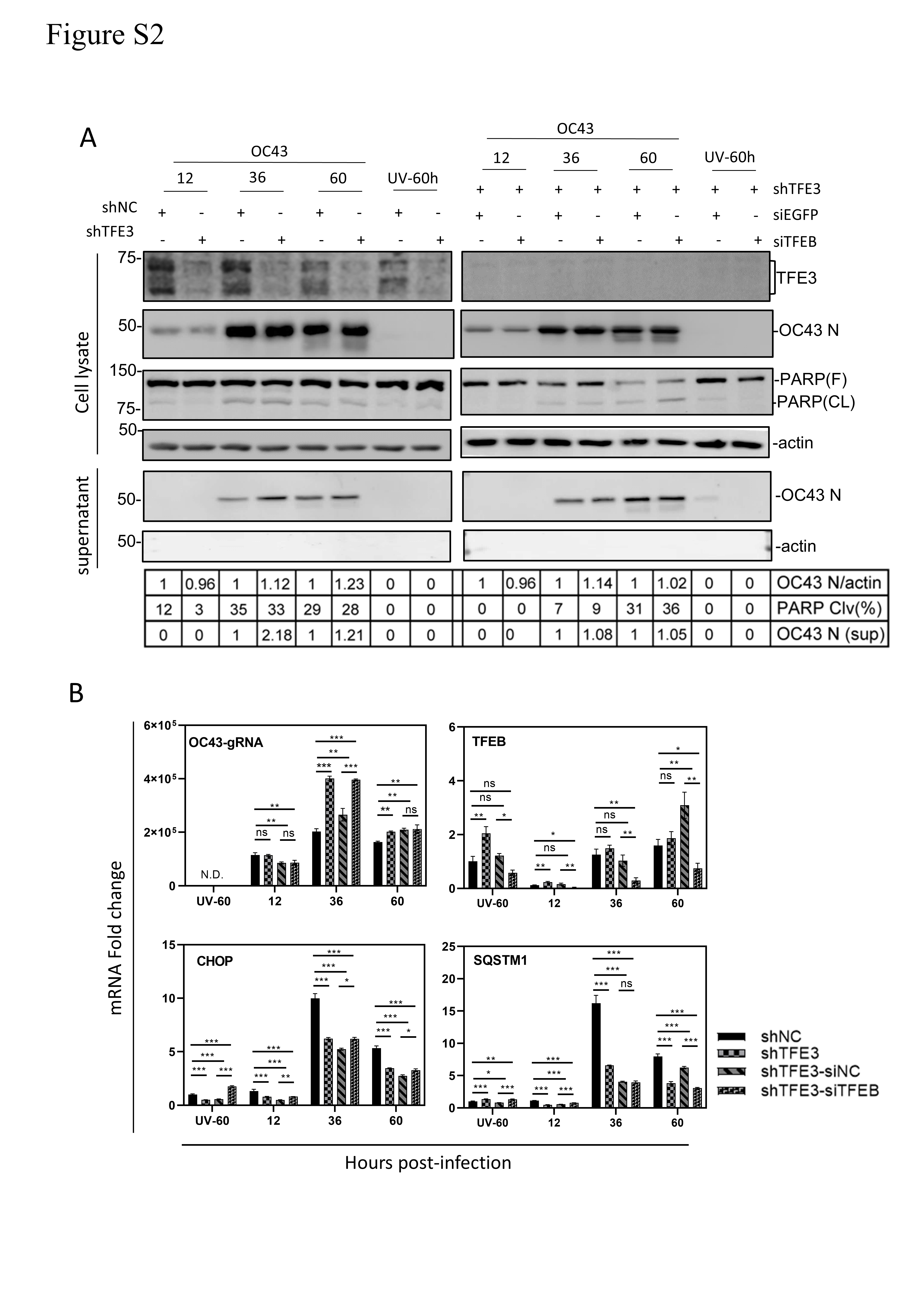

Supplement: Supplementary file 3 [file Image_2.tif]

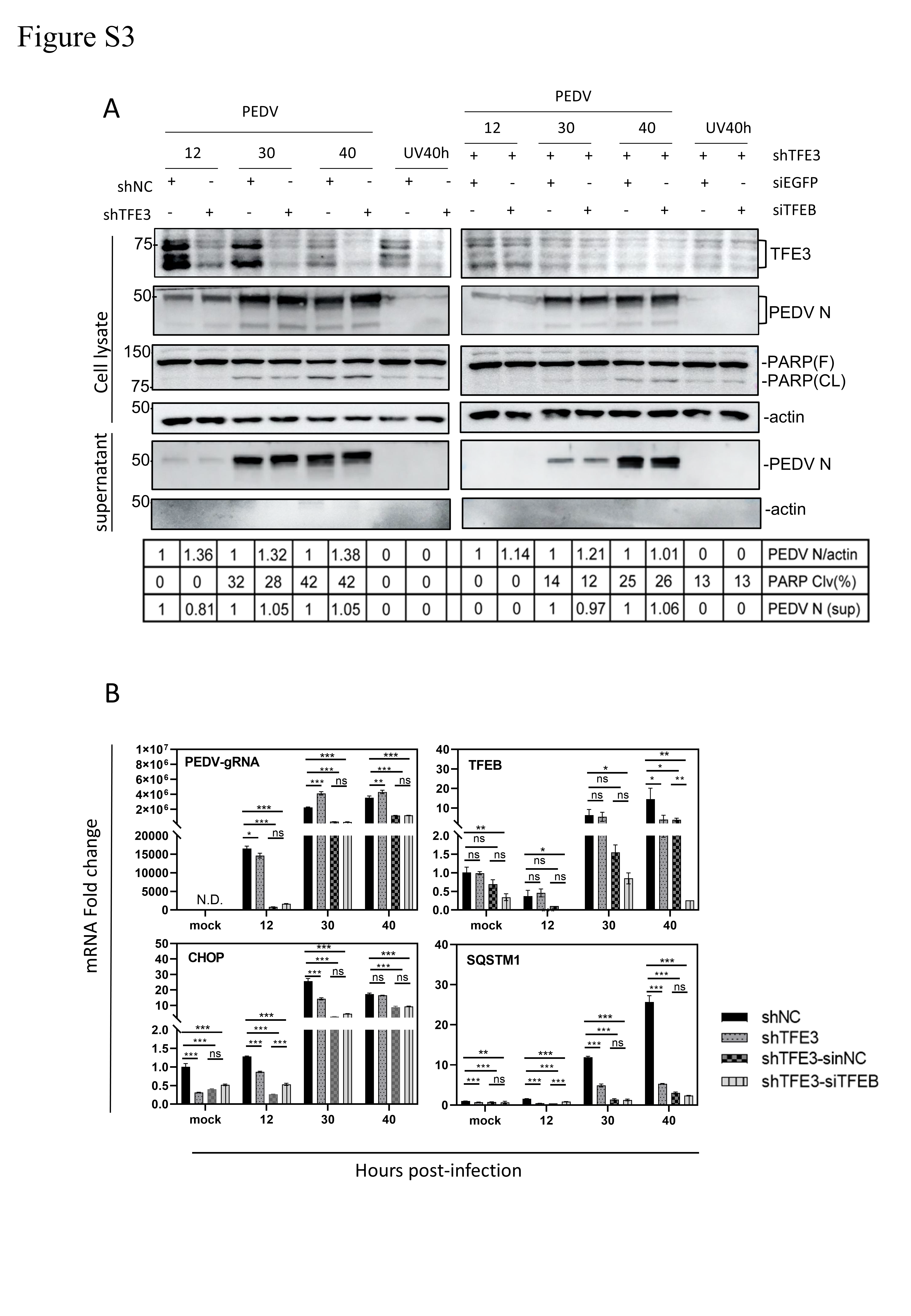

Supplement: Supplementary file 4 [file Image_3.tif]

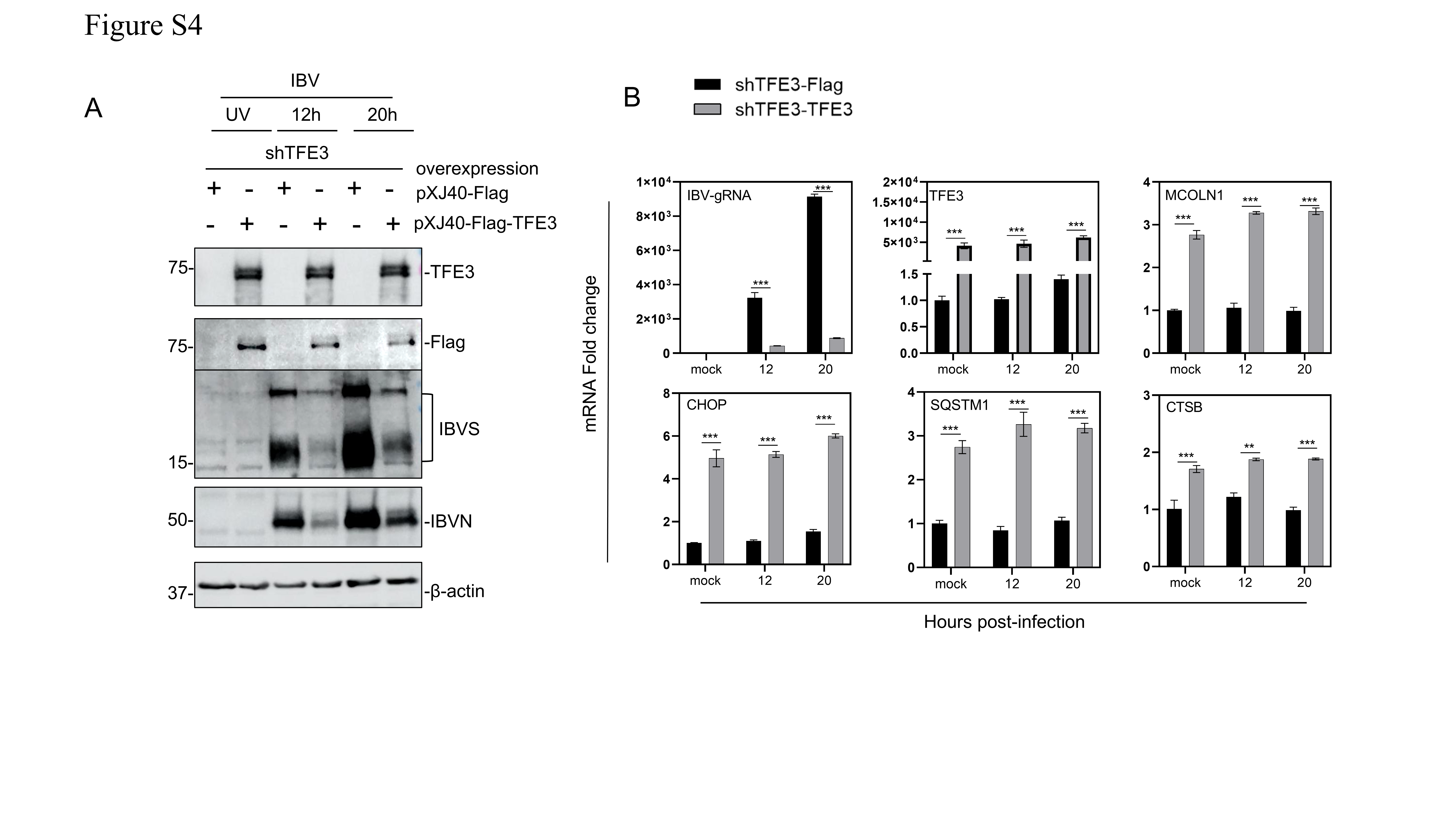

Supplement: Supplementary file 5 [file Image_4.tif]
